# Targeting Liver *Xor* by GalNAc-siRNA Is an Effective Strategy for Hyperuricemia Therapy

**DOI:** 10.3390/pharmaceutics16070938

**Published:** 2024-07-14

**Authors:** Huiyan Sun, Xinxia Wang, Yongqiang Li, Yingzhi Shen, Lin Zhang, Yingjie Xu, Junling Liu, Xuemei Fan

**Affiliations:** 1 Department of Biochemistry and Molecular Cell Biology, Shanghai Jiao Tong University School of Medicine, Shanghai 200025, China; 2 Shanghai Synvida Biotechnology Co., Ltd., Shanghai 201203, China

**Keywords:** hyperuricemia, GalNAc-siRNA, xanthine oxidoreductase

## Abstract

Hyperuricemia, i.e., increased plasma uric acid concentration, is a common problem in clinical practice, leading to gout or nephrolithiasis, and is associated with other disorders, such as metabolic syndrome, cardiovascular disease, and chronic renal disease. Xanthine oxidoreductase (XOR) is a critical rate-limiting enzyme involved in uric acid synthesis and a promising target for hyperuricemia therapy. However, XOR inhibitors currently face clinical problems such as a short half-life and side effects. Here, we found that specifically targeting liver *Xor* with GalNAc-siRNAs had a good therapeutic effect on hyperuricemia. First, siRNAs were designed to target various sites in the homologous region between *Homo sapiens* and *Mus musculus Xor* mRNA and were screened in primary mouse hepatocytes. Then, the siRNAs were modified to increase their stability in vivo and conjugated with GalNAc for liver-specific delivery. The effects of GalNAc-siRNAs were evaluated in three hyperuricemia mouse models, including potassium oxonate and hypoxanthine administration in *WT* and humanized *XDH* mice and *Uox* knockout mice. Febuxostat, a specific XOR inhibitor used for hyperuricemia treatment, was used as a positive control. Targeting liver *Xor* with GalNAc-siRNAs by subcutaneous administration reduced plasma uric acid levels, uric acid accumulation in the kidney, renal inflammation, and fibrosis, thereby alleviating kidney damage in hyperuricemia mouse models without hepatoxicity. The results demonstrated that targeting liver *Xor* with GalNAc-siRNAs was a promising strategy for hyperuricemia therapy.

## 1. Introduction

Hyperuricemia (HUA) is a common metabolic disease caused by increased production and/or underexcretion of uric acid. The prevalence of hyperuricemia continues to increase in a variety of regions, such as Australia and Ireland [1,2]. Long-term hyperuricemia can induce severe kidney damage, such as renal mitochondrial dysfunction, cortical oxidative stress, and tubular damage [3]. When uric acid concentration in blood is so high that monosodium urate (MSU) crystals form and accumulate, an inflammatory response in the host tissue occurs, thus resulting in intense pain from a gout attack [4]. Gout, which is usually described as intensive pain, swelling, warmth, and erythema, is one of the most prevalent inflammatory arthritic conditions worldwide and can even cause substantial limitations in physical movement, such as walking [5,6]. Uric acid is the final degradation product of purines, the main resources of which are catabolism of substances, food intake, and de novo synthesis. Hyperuricemia is often accompanied by other comorbidities, including cardiovascular disease [7], diabetic kidney disease [8], and chronic kidney disease (CKD) [9].

XOR is the rate-limiting enzyme in the process of uric acid production, catalyzing hypoxanthine to xanthine and subsequently xanthine to uric acid [10]. Compared with other mammals, some primates (for example, *Homo sapiens*) more easily develop hyperuricemia because of the loss of urate oxidase, which can convert uric acid to soluble allantoin [11]. Currently, there are three main strategies for treating hyperuricemia: reducing the production of uric acid, facilitating the excretion of uric acid, and converting uric acid into soluble allantoin. XOR inhibitors are popular in the clinical treatment of hyperuricemia, and many drugs targeting XOR, such as febuxostat and allopurinol, have been designed. However, there are many severe side effects associated with existing hyperuricemia treatments; for example, febuxostat increases the risk of cardiovascular mortality [12], while allopurinol can induce allopurinol hypersensitivity syndrome, with a mortality rate between 20% and 25% [13]. It has been reported that deletion of hepatocyte *Xdh* (the gene encoding XOR) significantly decreases liver and plasma uric acid concentrations [14]. Hence, targeting XOR in the liver to design new hyperuricemia drugs with high efficacy and few side effects to relieve patients from heavy economic burdens and severe physical and mental pain is promising.

In 2018, patisiran, the first siRNA drug approved by the U.S. Food and Drug Administration (FDA), was found to target transthyretin (TTR) messenger RNA in the liver to fight hereditary transthyretin amyloidosis [15], highlighting the potential of RNAi-based therapeutics for the treatment of diseases. siRNAs can bind to mRNAs at specific sites to regulate their degradation, thus controlling the expression of functional proteins; this process was first described by Andrew Fire and Craig Mello in 1998 [16]. Over the past several decades, with the development of chemical modification, sequence selection, and delivery innovation, a new pipeline of safer and more efficacious siRNA-based drugs has been created. Initially, appropriate chemical modifications, including reducing the activation of endogenous immune reactions [17], improving resistance to nuclease degradation, and mitigating off-target effects, played important roles in the translation of siRNA agents [18]. Moreover, the development of new drug delivery systems is essential for the development of new siRNA drugs. Currently, the delivery of siRNAs focuses on lipid nanoparticles (patisiran) [19] and N-acetylgalactosamine (GalNAc) (givosiran, lumasiran, and vutrisiran) [20]. In particular, the use of GalNAc-siRNA to target the liver is a well-defined method benefiting from its high affinity for the asialoglycoprotein receptor (ASGPR), which is abundant in the liver and has a high cycling speed [21]. Compared with antibodies or small molecule drugs, siRNAs have a shorter development span and a wider therapeutic area, especially for genes that are unfeasible for development via such strategies.

Here, we demonstrated the important role of liver XOR in causing high uric acid levels in vivo, and GalNAc-siRNA, which targets liver *Xor*, significantly protected the kidney from inflammation and fibrosis in hyperuricemia mouse models without hepatoxicity. Thus, we developed a novel therapeutic strategy for the treatment of hyperuricemia that is highly efficient and has few side effects.

## 2. Materials and Methods

### 2.1. Study Design

Firstly, siRNAs were designed to target various sites in the homologous region between *Homo sapiens* and *Mus musculus Xor* mRNA. The knock-down efficiency of siRNAs was evaluated in primary mouse hepatocytes (PMHs) by qPCR technology. In order to enhance the resistance of siRNAs to nuclease, chemical modification (ESC) was applied to naked-siRNAs, and GalNAc molecules were conjugated to achieve liver-specific targeting. Subsequently, GalNAc-siRNAs were administered subcutaneously to *WT* and humanized *XDH* (*hXDH)* mice with hyperuricemia induced by potassium oxonate and hypoxanthine (PO/HX), as well as *Uox*^−/−^ mice, and the efficacy and safety of the drugs were evaluated in vivo.

### 2.2. Preparation of the Models

This study was approved by the Institutional Animal Care and Use Committee (IACUC) of Shanghai Jiao Tong University School of Medicine. The ethical approval code is JUMC2023-093-A and the approval date is 20230719. All animal experiments strictly complied with ethical regulations for laboratory animals. All animals used were male C57BL/6J background mice of 8–12 weeks, unless otherwise noted.

#### 2.2.1. PO/HX-Induced Mouse Hyperuricemia Model

PO/HX combinative administration was used to induce hyperuricemia in *WT* or *hXDH* mice. Mice were given 300 mg/kg/b.w. potassium oxonate (PO; Sigma-Aldrich, 156124; St. Louis, MO, USA) intraperitoneally and 300 mg/kg/b.w. hypoxanthine (HX; Sigma-Aldrich, H9377) intragastrically for 21 consecutive days [22], except for the NC group. Mice in the NC group were given an equivalent volume of saline (i.p.) and 0.5% CMC-Na (i.g.) at the same time. Mice in the febuxostat group were administered febuxostat by gavage (5 mg/kg/b.w.; Yuanye Biotech., B27248; Shanghai, China) 1 h after the construction of the hyperuricemia model. For siRNA therapy, the GalNAc-siRNA group was subcutaneously administered 10 mg/kg/b.w. or 5 mg/kg/b.w. siRNA on day 0, 3, 7, and 14; the same dosing regimen can be found in publication [23]. The other groups, except the GalNAc-siX61 and GalNAc-siX60 groups, were subcutaneously administered an equivalent volume of saline. On the 21st day, the mice were sacrificed 1 h after drug treatment. Plasma and various organs were harvested, and the biochemical indices and pathological conditions of the mice were analyzed.

#### 2.2.2. The Construction of Uox^−/−^ Mice

The *Uox* knockout mice were generated by GemPharmatech Company Limited (Nanjing, China). Using CRISPR/Cas9 technology to knock out 2–4 exons of the wild-type allele, the background was C57BL/6J. Because of the loss of urate oxidase, uric acid accumulated in the body so that the mice suffered from hyperuricemia.

### 2.3. Construction Strategy of Humanized XDH Transgenic Mice

Human *XDH* transgenic mice were developed to integrate human *XDH* cDNA to replace the mouse *Xdh* gene by CRISPR/Cas9 technology by Shanghai Model Organisms. The human *XDH* exon 1–36 cDNA was knocked into the wild-type allele by homologous recombination, and a poly(A) transcription termination signal was generated after the human *XDH* cDNA was added to ensure that the mouse *Xdh* gene was completely replaced by the human *XDH* gene. The *XDH*-L-sgRNA sequence used was AGGCGTATCTTTCAAGTTGCAGG. The *XDH*-R-sgRNA sequence used was GAGTTGGTCTTCTTTGTGAATGG.

### 2.4. Primary Mouse Hepatocytes Isolation and Culture

Primary mouse hepatocytes were isolated using a standard two-step collagenase perfusion technique. All materials were sterilized in advance and the perfusion medium and digest medium were pre-warmed to 37 °C. After anesthesia, mice underwent laparotomy and portacaval intubation with the perfusion of 20 mL liver perfusion medium (Gibco, Thermo Fisher Scientific, Waltham, MA, USA) followed by 20 mL liver digest medium (Gibco) containing 0.05% collagenase type IV (Gibco). Then, the liver was removed and continued to be perfused with liver digest medium in a 10 cm dish, while gently pressing with a cotton swab. The liver cell suspension was collected, filtered through a 100 μM filter, and further purified with a density gradient centrifugation. The liver cells were seeded on 6-well plates, and siRNAs were transfected with Lipofectamine RNAiMAX (invitrogen, Thermo Fisher Scientific) after cell adhesion to the substrate. The cells were cultured in high-glucose DMEM (L110KJ, BasalMedia, Shanghai, China) with 10% FBS and 1% PS. All cells were incubated at 37 °C in a humidified environment with a CO_2_ concentration of 5%. At 48 h after transfection, cells were collected to obtain RNA, while at 120 h after transfection, protein samples were extracted from cells. All experiments with animals were approved by the Institutional Animal Care and Use Committee (IACUC) of Shanghai Jiao Tong University School of Medicine.

### 2.5. qPCR Analysis

Tissues such as the livers were cut into pieces and homogenized using a sample freezing grinder (Shanghai Jingxin, Shanghai, China). Cells were harvested at 48 h after transfection. Total RNA was obtained using TRIzol reagent (Vazyme Biotech, Nanjing, China), chloroform (Sinopharm Chemical Reagent Co., Ltd., Shanghai, China) was subsequently added, and isopropanol (Sinopharm Chemical Reagent Co., Ltd., China) was used to precipitate RNA in the supernatant after centrifugation. The total RNA was washed with 75% ethanol. Reverse transcription reagent (RR036A; Takara, Shiga, Japan) and a qPCR kit (RR820; Takara, Japan) were used to reverse transcribe equal amounts of RNA to cDNA [24,25], after which quantitative real-time polymerase chain reaction (qPCR) was performed. Gene expression was normalized to that of GAPDH. The primers used in the qPCR experiments were as follows: human *XOR*-Forward primer sequence, GAATAGGGTCGGGAAGGGTT; and human *XOR*-Reverse primer sequence, CACAGGAAGGCACACGATTT. The human *GAPDH* forward primer sequence was CATCACCATCTTCCAGGAG, and the human *GAPDH* reverse primer sequence was AGGCTGTTGTCATACTTCTC. The mouse *Xor* forward primer sequence was ATTTGGCAGCATCCCCATTG, and the mouse *Xor* reverse primer sequence was GTTTGGCGTTACTGTCTCCG. The mouse *Gapdh* forward primer sequence was AGGTCGGTGTGAACGGATTTG, and the mouse *Gapdh* reverse primer sequence was TGTAGACCATGTAGTTGAGGTCA.

### 2.6. Preparation of GalNAc-siRNAs

The modified siRNA sequences were synthesized by the solid-phase phosphoramidite method using a nucleic acid synthesizer (Dr. Oligo 48 synthesizer, Biolytic Lab Performance, Inc., Fremont, CA, USA) [26]. The synthesis process includes deblocking, coupling, capping, and oxidation. Universal CGPs were selected as solid-phase carriers. GalNAc, synthesized by Shanghai Synvida Biotechnology Company (Shanghai, China), was added to the synthesis column before initiating the synthesis process to produce GalNAc-sense strands. All modified nucleotide monomers were purchased from ChemGenes Corporation (Wilmington, MA, USA). siRNA synthesis required the use of concentrated ammonia water and heating to 70 °C for 3 h. After elution from the synthetic columns, siRNAs were purified using OPC columns, HPLC, or PAGE. After that, the siRNAs were dried into powder using a vacuum dryer (Beijing JM Technology Co., Ltd., Beijing, China) and stored at −20 °C. The sense strands and antisense strands were mixed equally and annealed at 94 °C for 3 min to combine into siRNAs. UPLC-TOF/MS and agarose gel electrophoresis were used to confirm the purity of the siRNAs. The purity of GalNAc-siRNAs exceeded 85% as determined by UPLC-TOF/MS analysis.

### 2.7. siRNA Stability and Purity Measurement

A total of 50 µg/mL ribonuclease A (10406ES03, Yeasen, Shanghai, China) and normal human plasma were used to detect siRNA stability. siRNAs were mixed with ribonuclease A in equal volumes and incubated at 37 °C or mixed with plasma and incubated. The mixture of siRNA and ribonuclease A was collected at 0, 10, and 60 min, while the mix of siRNA and plasma was harvested at 0, 10, 24 h, followed by the addition of siRNA loading buffer immediately. Agarose gels (4%) and polyacrylamide gel electrophoresis (20% PAGE) were prepared in 1× TBE buffer and stained with YeaRed nucleic acid gel stain (10202ES76, Yeasen, China). A 1× TBE buffer was also used as the running buffer. The samples were loaded in the agarose gel by electrophoresis at 180 V for 30 min. Subsequently, the siRNA bands were analyzed under UV illumination using a Tanon 1600 Gel Imaging System (Tanon, Shanghai, China). This method was also used to analyze the purity of the GalNAc-siRNAs.

### 2.8. Western Blot Analysis

Tissues or cells were collected and lysed with protease inhibitor to extract total protein. Tissues such as the liver and kidney were cut into pieces and homogenized using a sample freezing grinder (Shanghai Jingxin, Shanghai, China). The samples were subsequently centrifuged at 4 °C and 12,000× *g* for 30 min. The total protein concentration was determined via a BCA kit (20201ES86, Yeasen, China). Amounts of 8% or 10% SDS-PAGE were used to separate proteins with different molecular weights. After that, the proteins were transferred to a PVDF membrane. Five percent skim milk was used to block the membrane for 1 h at room temperature. Afterward, the proteins were incubated overnight at 4 °C with primary antibodies. The next day, after three washes with TBST, the membrane was incubated with secondary antibodies at room temperature for 1 h, followed by the last step of ECL addition for exposure. The antibodies used were anti-XOR antibody (sc-398548; Santa Cruz Biotechnology, Dallas, TX, USA; 1:1 K), anti-HSP90 (13171-1-AP; Proteintech, Wuhan, China; 1:1 K), anti-GAPDH (30201ES; Yeasen; 1:10 K), anti-rabbit IgG secondary antibody (111-035-003; Jackson ImmunoResearch, West Grove, PA, USA; 1: 10 K), and anti-mouse IgG secondary antibody (115-035-003; Jackson ImmunoResearch, USA; 1:5 K).

### 2.9. Biochemical Assay

The uric acid concentrations of the plasma and tissue homogenates were measured with standard kits according to the manufacturer’s instructions (Jiancheng Bioengineering Institute, Nanjing, China). ALT, AST, CRE, and BUN levels were analyzed via an automatic biochemical analyzer (JYK-SH-AU5800, Beckman Coulter, Brea, CA, USA). The concentrations of inflammatory cytokines in mice plasma after subcutaneous administration of 10 mg/kg GalNAc-siRNAs were detected using ELISA kits (Shanghai Enzyme-linked Biotechnology Co., Ltd., Shanghai, China).

### 2.10. Histopathological Assessment

The liver and kidney tissues of the mice were collected, fixed in 4% paraformaldehyde for 48 h, dehydrated in alcohol step by step (75% alcohol→85% alcohol→95% alcohol I→95% alcohol II→100% alcohol I→100% alcohol II), and then soaked in xylene (xylene I→xylene II). After being embedded in paraffin, the tissues were sectioned into 4 µm sections (Leica RM2235, Wetzlar, Germany). Hematoxylin and eosin (HE) staining and Masson staining were performed. The stained slices were observed under a light microscope (Zeiss, Oberkochen, Germany) and photographed. The renal sections were scanned entirely using a high-throughput slide scanner (Axio Scan. Z1, Zeiss, Oberkochen, Germany). ImageJ (Java 1.8.0_345) was used to quantify the lesion area (area of collagen deposition (%) = average collagen area/area of total field × 100).

### 2.11. Hematoxylin and Eosin (HE) Staining

Paraffin-embedded tissues were cut into 4 µm slices and pasted on a microscope slide. The slices were soaked in xylene and alcohol to remove paraffin. Then, the slices were stained with hematoxylin (BA4097, Baso, Zhuhai, China) for 3 min. Ethanolic hydrochloric acid and water were used after hematoxylin. Eosin (BA4098, Baso, China) was applied to stain slices for 1 min, followed by dehydration in alcohol and clearing in xylene. Last but not least, slices were sealed with neutral balsam (36313ES60, Yeasen, China) in preparation for microscopy imaging.

### 2.12. Masson Staining

Paraffin-embedded tissues were cut into 4 µm slices and pasted on a microscope slide. The slices were stained with hematoxylin (517-28-2; Sangon, Shanghai, China) for 8 min (the staining time was adjusted according to the different tissues; hematoxylin A solution was mixed with B solution in an equal volume and used within 4 h), followed by the addition of distilled water to remove excess dye solution. Then, the slices were immersed in ethanolic hydrochloric acid and rinsed under flowing water to reverse blue. After being dyed with Ponceau 2R (A600753, Sangon, China) and fuchsin acid (A610469, Sangon, China) for 8 min, the slices were washed with 0.2% acetic acid solution and differentiated with 5% phosphomolybdic acid (A600709, Sangon, China) for 15 min, followed by two washes with 0.2% acetic acid solution. The slices were dyed with 0.1% brilliant green (A610007, Sangon, China) for 5 min. A 0.2% acetic acid solution was used to remove excess dye. Finally, the slices were sealed, viewed under a light microscope, and photographed.

### 2.13. Statistical Analysis

For the statistical analyses of the experimental results, Prism 8 software was used. Two-tailed unpaired Student’s *t* test was used for comparisons between two groups. Comparisons among multiple groups were performed using one-way ANOVA followed by Tukey’s multiple comparison test. The data are shown as the mean ± standard deviation (SD). In all the data comparisons, a *p* value less than 0.05 was considered to indicate statistical significance.

## 3. Results

### 3.1. Screening of XOR siRNAs with High Efficiency in Primary Mouse Hepatocytes

**Figure 1:**
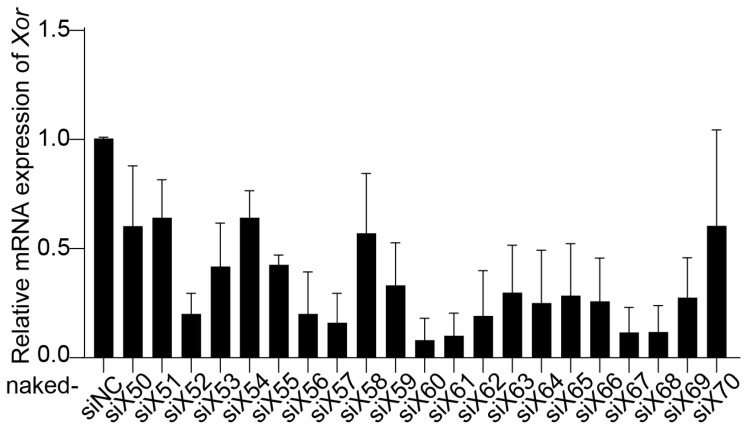
Screening of *Xor* siRNAs with high efficiency in primary mouse hepatocytes (PMHs). Twenty-one pairs of siRNAs (naked-siX50 to naked-siX70) were designed to target various sites in the homologous region between *Homo sapiens XOR* mRNA and *Mus musculus Xor* mRNA. The silencing efficiency of the siRNAs was evaluated by qPCR in primary mouse hepatocytes (PMHs) after transfection with different *Xor* siRNAs (10 nM) for 48 h. The data are shown as the fold change versus naked-siNC. *n* = 3 independent experiments.

Twenty-one pairs of siRNAs (naked-siX50 to naked-siX70) were designed to target various sites in the homologous region between *Homo sapiens XOR* mRNA and *Mus musculus Xor* mRNA. The inhibitory effect of siRNAs on XOR expression was evaluated in primary mouse hepatocytes (PMHs). PMHs were isolated from C57BL/6J mice using a two-step collagenase perfusion technique [27], and naked-siRNAs were transfected with Lipofectamine RNAiMAX. Compared with the negative control siRNA (naked-siNC), naked-siX60, naked-siX61, naked-siX67, and naked-siX68 had the highest efficiency in knocking down *Xor* mRNA expression among the 21 pairs of siRNAs (Figure 1).

**Figure 2:**
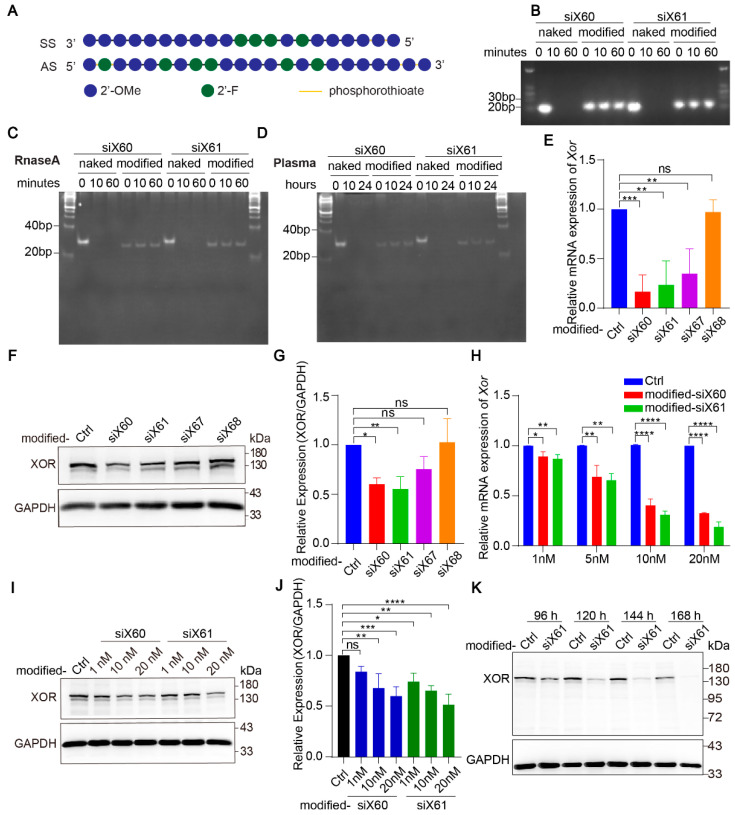
Modified-siRNAs showed great stability and silencing efficiency. (**A**) The *Xor* siRNAs were modified with the “Advanced ESC” strategy. Abbreviations: 2’-F, 2’-fluoro; 2’-OMe, 2’-methoxy; SS, sense strand; AS, antisense strand. (**B**) Gel electrophoresis of naked and modified *Xor* siRNAs after coincubation with nuclease for 0, 10, or 60 min. (**C**,**D**) siRNAs after coincubation with nuclease for 0, 10, or 60 min (**C**) or human plasma for 0, 10, 24 h (**D**). Twenty percent polyacrylamide gel electrophoresis (PAGE) was used to separate nucleic acids with different molecular weight. (**E**–**G**) The silencing efficiency of modified siRNAs was evaluated in PMHs by qPCR after transfection for 48 h (**E**) or by Western blot (**F**) after transfection for 120 h with different modified siRNAs (10 nM). The relative expression of the XOR protein was quantified and calculated as the ratio of XOR/GAPDH, shown as the fold change versus the PBS control (**G**). (**H**) *Xor* mRNA expression was tested by qPCR after PMHs were transfected with modified-siX60 or modified-siX61 at the indicated concentrations (1, 5, 10, or 20 nM) for 48 h. Relative *Xor* mRNA expression is shown as the fold change versus the PBS control. (**I**,**J**) XOR protein levels were tested by Western blotting after PMHs were transfected with modified-siX60 or modified-siX61 at the indicated concentrations (1, 10, or 20 nM) for 120 h. GAPDH was used as a loading control. The relative XOR protein expression was calculated as the XOR/GAPDH ratio and is presented as the fold change versus the PBS control (**J**). (**K**) Representative Western blot images of the XOR protein after PMHs were transfected with modified-siX61 for 96, 120, 144, or 168 h (20 nM). GAPDH was used as a loading control. The results are presented as the mean ± SD; ns, *p* > 0.05; * *p* < 0.05, ** *p* < 0.01, *** *p* < 0.001, **** *p* < 0.0001. *n* = 3 independent experiments.

Naked-siRNA is easily degraded by nucleases in blood. To improve the stability of the siRNAs and their delivery efficiency in vivo, the siRNAs were modified with enhanced stabilization chemistry (ESC) (Figure 2A). At 10 min after the coincubation of nuclease, the naked-siRNAs were completely degraded, while the modified-siRNAs were resistant to nuclease even after 60 min (Figure 2B,C). In plasma, the naked-siRNAs were completely degraded after 10 h, while the modified-siRNAs were undamaged after 24 h (Figure 2D). After modification, siX60 and siX61 had high *Xor* silencing efficiency, while the silencing efficiency of siX67 and siX68 was significantly reduced (Figure 2E–G). Compared with those in the control group, the expression of *Xor* mRNA and protein was inhibited by modified-siX60 and modified-siX61 in a concentration- and time-dependent manner (Figure 2H–K).

**Figure 3:**
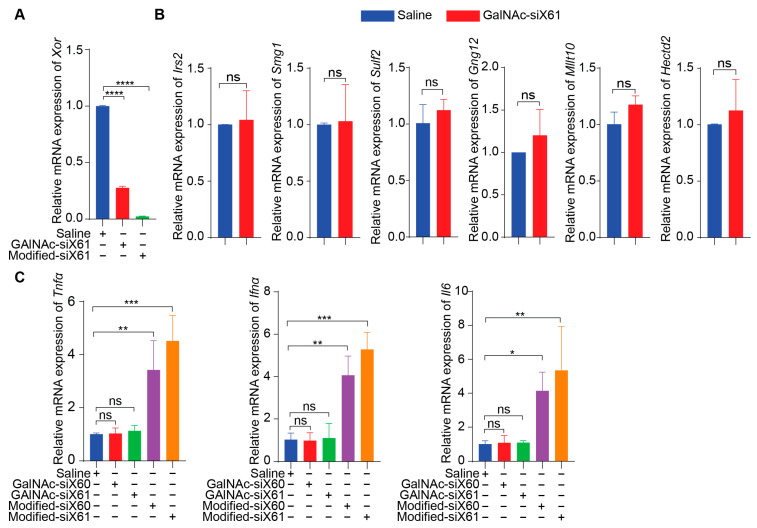
The safety profile of PMHs after coincubation with GalNAc-siX61 or transfection with modified-siX61. (**A**) The silencing efficiency of GalNAc-siX61 and transfected modified-siX61 was tested in PMHs by qPCR after transfection for 48 h (20 nM). Modified-siX61 was transfected with lipofectamine. (**B**) Verification of off-target effect of GalNAc-siX61. Six genes that are highly expressed in liver and have similarity greater than 95% with siX61 were selected to detect siRNA off-target effects. The relative mRNA expression of *Hectd2*, *Irs2*, *Smg1*, *Mllt10*, *Gng12*, and *Sulf2* in primary mouse hepatocytes was detected by qPCR at 48 h after GalNAc-siX61 administration (20 nM). (**C**) The mRNA expression of inflammatory cytokines containing *Tnfα*, *Ifnα*, and *Il6* in PMHs after coincubation with GalNAc-siX61 or transfected modified siRNA with lipofectamine were tested by qPCR (20 nM). The results are presented as the mean ± SD; ns, *p* > 0.05; * *p* < 0.05, ** *p* < 0.01, *** *p* < 0.001, **** *p* < 0.0001. *n* = 3 independent experiments.

To specifically target *Xor* in the liver, a GalNAc delivery system was used. GalNAc-siX60 and GalNAc-siX61 were obtained by conjugating modified siX60 and siX61 with synthetic tri-antennary GalNAc. The results in Figure 3A showed that GalNAc-siRNAs could be ingested by PMHs and inhibit the expression of *Xor* in PMHs. According to the sequence of siX61, six genes that were highly expressed in the liver among sequences with similarity greater than 95% were selected to detect siRNA off-target effects. The relative expression of *Hectd2*, *Irs2*, *Smg1*, *Mllt10*, *Gng12,* and *Sulf2* in primary mouse hepatocytes at 48 h after GalNAc-siX61 administration was measured by qPCR. The results demonstrated that the mRNA expression of these genes was not affected by GalNAc-siX61 (Figure 3B), indicating no notable off target effects. Furtherly, the mRNA expression of inflammatory cytokines containing Tnfα, Ifnα, and Il6 in PMHs after coincubation with GalNAc-siX61 or transfected modified siRNA with lipofectamine were tested by qPCR (Figure 3C). The results showed that transfected modified siRNA with lipofectamine stimulated immune response indeed; however, GalNAc-siRNA did not.

### 3.2. GalNAc-siX61 Had Liver-Specific Targeting and Long-Term Effect In Vivo

**Figure 4:**
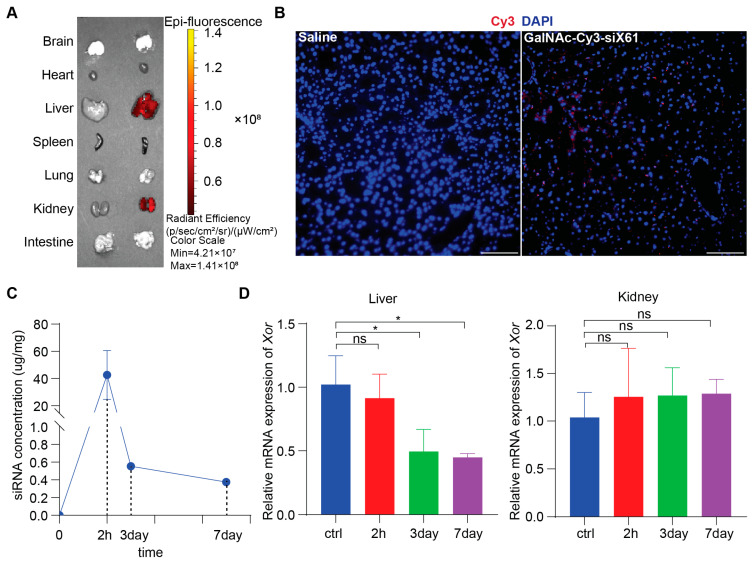
The biodistribution and pharmacokinetic studies of GalNAc-siX61 in wild type C57BL/6J male mice. (**A**) Fluorescence signal of isolated organs was captured by IVIS SpectrumCT at 72 h after s.c. with 10 mg/kg Cy3-labeled- GalNAc-siX61 or equal volume saline. Representative images are shown. (**B**) Representative images of immunofluorescence staining on liver sections from saline group mice, GalNAc-Cy3-siX61 group mice for DAPI (blue), and GalNAc-Cy3-siX61 (red). Scale bar, 100 μm. (**C**) Concentration–time profiles of GalNAc-siX61 in liver after a single s.c. administration of 10 mg/kg. *n* = 3 independent experiments. (**D**) Relative mRNA expression of *Xor* in liver or kidney at 2nd hour, 3rd day, or 7th day after mice were given a single dose of 10 mg/kg GalNAc-siRNAs. The results are presented as the mean ± SD; ns, *p* > 0.05; * *p* < 0.05. *n* = 3 independent experiments.

To study in vivo biodistribution, various organs from wild type C57BL/6 male mice after administering 10 mg/kg GalNAc-Cy3-siX61 or equal volume saline at 72 h were collected and images were captured by IVIS SpectrumCT (PerkinElmer, Shelton, CT, USA). The results showed that GalNAc-siRNAs mainly distributed in the liver and kidney, without existence in other organs (Figure 4A). Images of immunofluorescence staining showed accumulation of GalNAc-Cy3-siX61 in the liver (Figure 4B). Subcutaneous delivery of GalNAc-siRNA results in rapid absorption by hepatocytes, leading to the short half-life of GalNAc-siRNA in plasma for several hours [28]. Therefore, in order to evaluate the pharmacokinetics of GalNAc-siX61, the concentrations of GalNAc-siX61 in the liver were measured by qPCR. After a single 10 mg/kg dose of GalNAc-siX61 to wild type C57BL/6J male mice, a mean peak concentration (C_max_) was observed at 2 h after administration and GalNAc-siX61 reached a low concentration in the liver at day 7 (Figure 4C). The knock-down effect began at day 3 and reached its maximum at day 7, showing a delayed effect compared to the drug concentration in the liver (Figure 4D), which conforms to the general metabolic law of GalNAc-siRNAs [28].

### 3.3. GalNAc-siRNAs Reduced Xor Expression in Liver and Lowered Uric Acid Levels in Plasma

**Figure 5:**
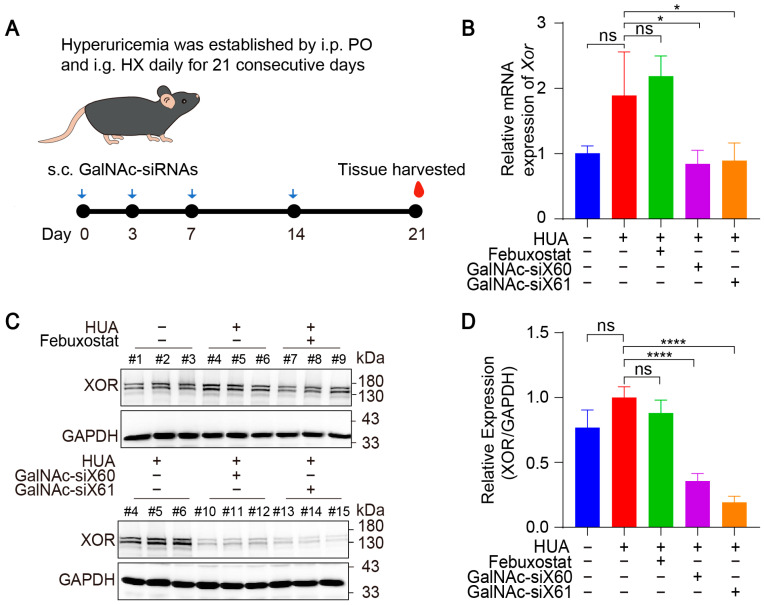
GalNAc-siRNAs reduced *Xor* expression in liver in vivo. (**A**) Schematic diagram of drug administration to *WT* mice. Hyperuricemia was induced in mice via intraperitoneal injection (i.p.) of potassium oxonate (PO) and intragastric administration (i.g.) of hypoxanthine (HX) daily for 21 days. Febuxostat was administered daily via i.g. and was used as a positive control, while GalNAc-siRNAs were administered via subcutaneous injection (s.c.) at 10 mg/kg on days 0, 3, 7, and 14. Blood and tissues were harvested on day 21 and measured. (**B**) The relative mRNA expression of *Xor* in liver was assessed by qPCR on day 21. (**C**) Representative images of western blots showing XOR protein expression in liver on day 21. GAPDH was used as a loading control. (**D**) Relative expression of XOR protein in (**C**) was quantified and calculated as the ratio of XOR/GAPDH. The results are presented as the mean ± SD; ns, *p* > 0.05; * *p* < 0.05, **** *p* < 0.0001. *n* = 3 independent experiments.

To evaluate the in vivo therapeutic effect of GalNAc-siRNAs, hyperuricemic mice were established by injecting potassium oxonate (PO) and hypoxanthine (HX) for 21 consecutive days [22]. Due to the short life span [29,30], the febuxostat group was intragastrically administered febuxostat each day after 1h construction of hyperuricemia mice, and the other groups were intragastrically administered saline every day. The GalNAc-siX60 and GalNAc-siX61 groups were subcutaneously administered GalNAc-siRNAs on days 0, 3, 7, and 14, the same dosing regimens as publication [23], while the other groups were subcutaneously administered the same volume of saline (diagram shown in Figure 5A). All animals were euthanized, and blood and tissues were harvested 1 h after gavage on day 21.

**Figure 6:**
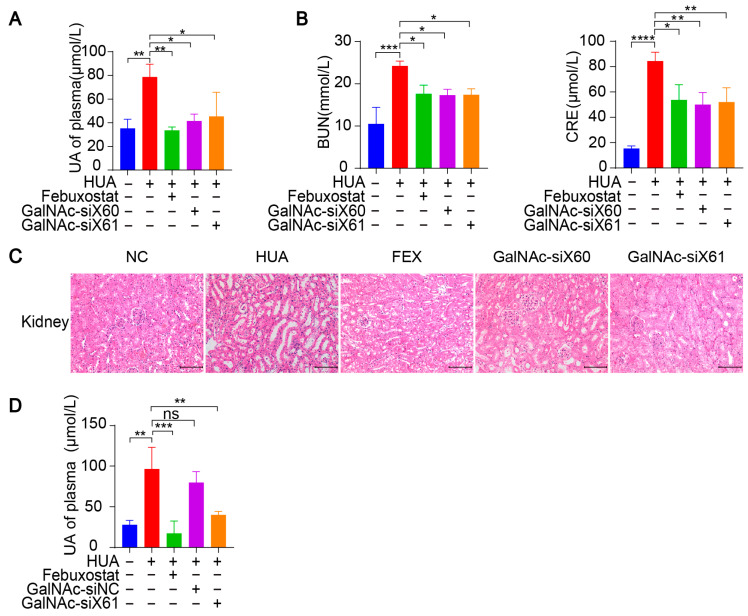
GalNAc-siRNAs lowered uric acid levels in plasma and alleviated kidney damage in *WT* mice. (**A**–**C**) Hyperuricemia was induced in *WT* mice as per the diagram in Figure 5A. Blood and tissues were harvested on day 21 and measured. The uric acid (UA) level in plasma was tested (**A**). Indicators of kidney damage in plasma, including blood urea nitrogen (BUN) and creatinine (CRE), were measured (**B**). Representative images of HE-stained kidneys (**C**). Scale bar, 100 μm. (**D**) Hyperuricemia was induced in *WT* mice via the diagram in Figure 5A. GalNAc-siNC was used as a negative control for GalNAc-siX61. The UA level of plasma was tested. The results are presented as the mean ± SD; ns, *p* > 0.05; * *p* < 0.05, ** *p* < 0.01, *** *p* < 0.001, **** *p* < 0.0001. *n* = 3 independent experiments.

The mRNA and protein expression levels of liver *Xor* were decreased significantly after GalNAc-siX60 or GalNAc-siX61 treatment (Figure 5B–D). Febuxostat did not affect *Xor* expression, which was consistent with the findings of previous reports [31]. Mice showed an increase in plasma uric acid levels when treated with PO and HX, which was significantly reduced by febuxostat, GalNac-siX60, and GalNac-siX61 (Figure 6A).

Since the kidney is the main organ that excretes uric acid, large amounts of uric acid accumulate in the kidney during hyperuricemia, leading to kidney damage [32]. Hyperuricemia is now considered an independent risk factor for the occurrence and development of diabetic nephropathy, acute kidney injury, chronic kidney disease, and end-stage renal disease [33]. Compared with those in control group, the serum blood urea nitrogen (BUN) and creatinine (CRE) levels, which are important indicators of kidney injury, were significantly greater after PO and HX treatment. However, the BUN and CRE levels were reduced by febuxostat, GalNAc-siX60, or GalNAc-siX61 treatment (Figure 6B). HE staining revealed severe kidney damage after PO and HX treatment, which included renal tubule swelling, proximal tubule necrosis and dilation, indistinct boundaries between adjacent proximal tubule cells, and cytoplasmic vacuolation. Kidney damage was significantly alleviated by febuxostat, GalNAc-siX60, or GalNAc-siX61 treatment (Figure 6C).

Moreover, a scrambled sequence named GalNAc-siNC was synthesized and used as a negative control for GalNAc-siX61. The results showed that febuxostat and GalNAc-siX61 had a good effect in reducing serum UA. However, GalNAc-siNC did not mitigate the hyperuricemia condition (Figure 6D). The data demonstrated that the role of GalNac-siX61 in hyperuricemia therapy was due to its effect on XOR knock-down instead of non-specific effects of GalNAc. These data indicated that targeting liver *Xor* via GalNAc-siRNAs was an effective and long-acting strategy for hyperuricemia therapy.

### 3.4. Inhibition of Liver XOR Expression by GalNAc-siRNAs Reduced Plasma Uric Acid Levels and Alleviated Kidney Damage in Humanized XDH Mice

**Figure 7:**
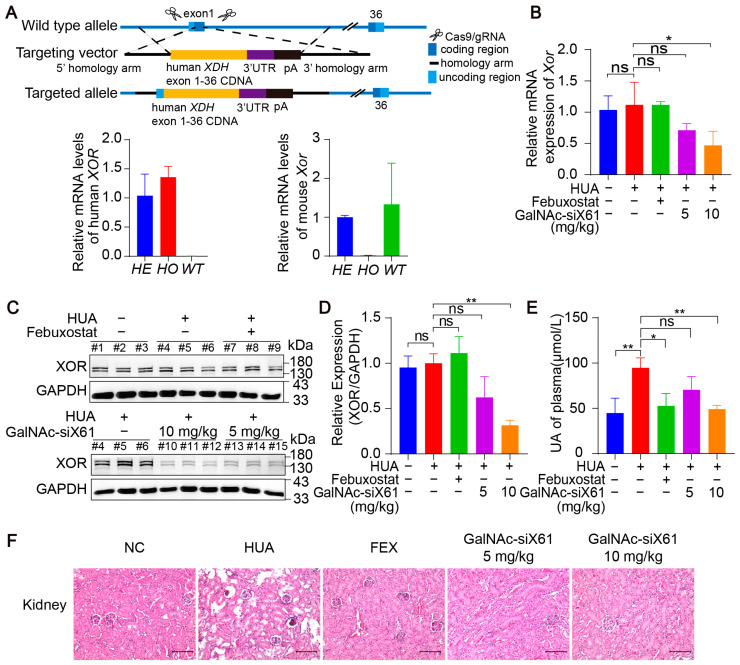
GalNAc-siRNAs reduced plasma uric acid levels and alleviated kidney damage in humanized *XDH* mice. (**A**) Schematic diagram illustrating the construction of humanized *XDH* mice and induction of hyperuricemia according to the workflow represented in Figure 5A. (**B**) The relative mRNA expression of *Xor* in liver was assessed by qPCR on day 21. (**C**) Representative images of Western blots showing XOR protein expression in liver on day 21. GAPDH was used as a loading control. (**D**) Relative expression of XOR protein in (**C**) was quantified and calculated as the ratio of XOR/GAPDH. (**E**) The uric acid (UA) concentration in the plasma was tested on day 21. (**F**) Representative images of HE-stained kidneys. Scale bar, 100 μm. The results are presented as the mean ± SD. ns; *p* > 0.05; * *p* < 0.05, ** *p* < 0.01. *n* = 3 independent experiments.

To investigate the efficacy of these GalNAc-siRNAs in targeting human *XOR*, humanized *XDH* mice were generated. Using CRISPR/Cas9 technology, the human *XOR* expression frame was tapped into the exon 1 site of the C57BL/6J *Xdh* gene through homologous recombination (Figure 7A). All the humanized *XDH* mice were treated as shown in Figure 5A. Compared with that in the control group, the liver *XOR* expression was noticeably decreased by GalNAc-siX61 both on the mRNA and protein levels in a concentration-dependent manner (Figure 7B–D). Moreover, GalNAc-siX61 reduced the plasma uric acid level and mitigated kidney damage in humanized *XDH* mice with hyperuricemia (Figure 7E,F). These data indicated that GalNAc-siRNAs were also effective at targeting human *XOR.*

### 3.5. Inhibition of Liver Xor Expression by GalNAc-siRNAs Relieves Kidney Damage in a Uox^−/−^ Mouse Model

**Figure 8:**
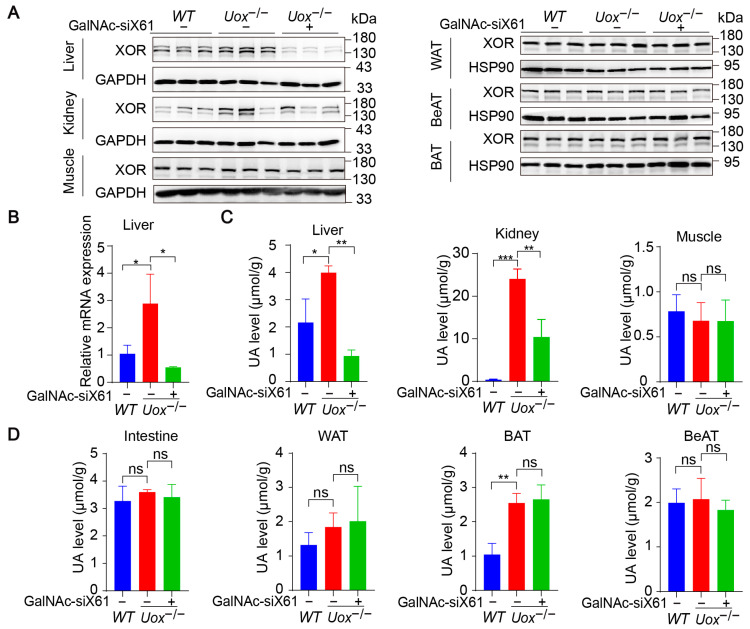
GalNAc-siX61 specifically silenced liver *Xor* expression in the *Uox*^−/−^ mouse model. GalNAc-siX61 (10 mg/kg) was administered on days 0, 3, 7, 14, 21, and 35, while the *WT* and control groups were given an equal volume of saline by subcutaneous injection. All samples were collected on day 45. Male *Uox*^−/−^ mice of 13–14 weeks were used. (**A**) Representative Western blot images of the XOR protein in liver, kidney, muscle, white adipose tissue (WAT), brown adipose tissue (BAT), and beige adipose tissue (BeAT) on day 45. GAPDH was used as a loading control. (**B**) The relative mRNA expression of *Xor* in liver was assessed by qPCR and is shown as the fold change versus *WT*. (**C**,**D**) The uric acid (UA) level was measured in liver, kidney, muscle, intestine, WAT, BAT, and BeAT. ns, *p* > 0.05; * *p* < 0.05, ** *p* < 0.01, *** *p* < 0.001. *n* = 3 independent experiments.

**Figure 9:**
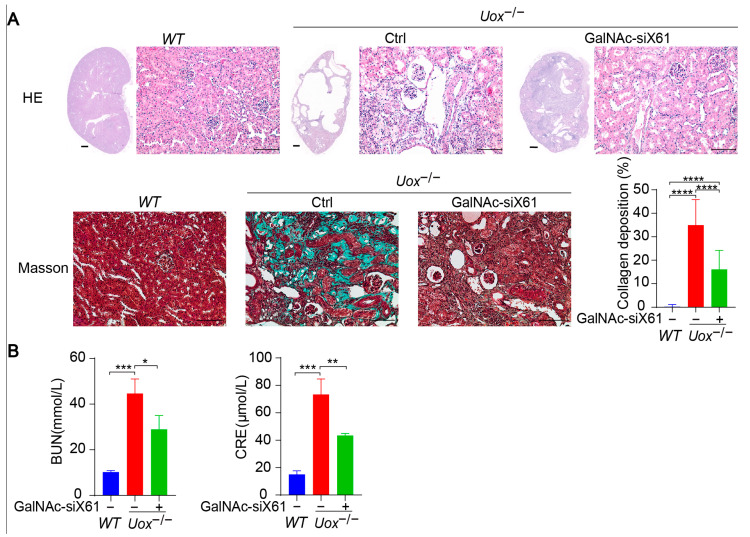
Inhibition of liver XOR expression by GalNAc-siRNAs relieved kidney damage in *Uox*^−/−^ mouse model. (**A**) Representative images of HE staining and Masson staining of kidneys from mice in the *WT*, control, and GalNAc-siX61 groups. HE staining of whole-kidney longitudinal sections; scale bar for whole- kidney image, 500 μm; scale bar for enlarged image, 100 μm. Green indicates collagen deposition. The collagen area (green) was calculated. (**B**) Indicators of kidney damage in plasma, including BUN and CRE, were measured. * *p* < 0.05, ** *p* < 0.01, *** *p* < 0.001, **** *p* < 0.0001. *n* = 3 independent experiments.

The urate oxidase gene (*Uox*) encodes uricase, which degrades uric acid into allantoin. The loss of uricase in humans during primate evolution increases vulnerability to hyperuricemia [34,35]. Therefore, *Uox* knockout mice (*Uox*^−/−^) were used as a “human-like” model to study hyperuricemia [34]. At 4 weeks of age, *Uox*^−/−^ mice had a high mortality rate of 65% because of severe nephropathy (including uric acid crystal deposition, multiple cysts, tubular atrophy, and collapse of the nephron) [36]. On the sixth day after birth, urate crystals could be observed in the kidney, while, beginning on the fourteenth day, urate crystals began to resolve, and hydronephrotic lesions appeared [36,37]. After GalNAc-siX61 treatment, the mRNA and protein expression levels of *Xor* and uric acid were significantly lower in the liver than in other tissues, including intestine and adipose tissue, which were reported to express *Xor* (Figure 8A,B). These results demonstrated the high specificity of GalNAc-siX61 for targeting liver *Xor*. The increase in uric acid levels in the kidney was significantly reduced by GalNAc-siX61 (Figure 8C,D). Moreover, fibrosis, inflammation, and damage to the kidney were strongly alleviated by GalNAc-siX61 (Figure 9A,B). These data demonstrated that targeting liver *Xor* with GalNAc-siRNA was a promising strategy for hyperuricemia therapy.

### 3.6. GalNAc-siX61 Showed No Off-Target Effects, Immunogenicity Reactions, or Toxicity In Vivo

**Figure 10:**
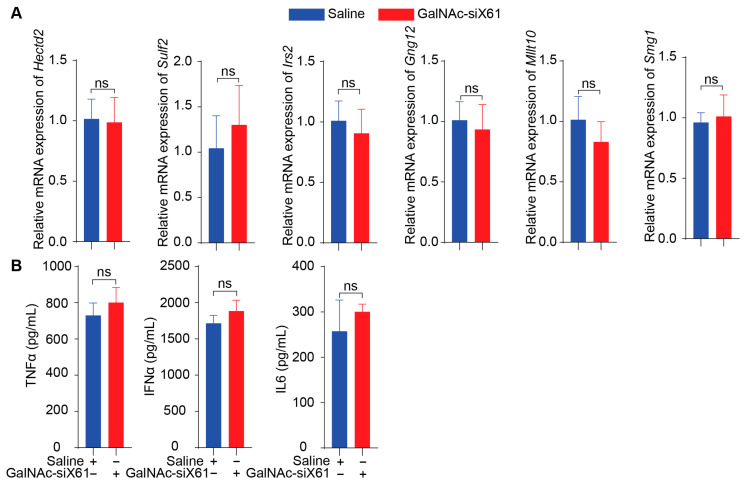
GalNAc-siX61 did not bring about off-target effects or immunogenicity reactions in vivo. (**A**) Six genes that are highly expressed in liver and have similarity greater than 95% with siX61 were selected to detect siRNA off-target effects. QPCR was used to evaluate their relative mRNA expression. (**B**) ELISA assay determined the serum level of TNFa, IL6, and IFNa in C57BL/J male mice treated with saline or GalNAc-siX61. ns, *p* > 0.05. *n* = 3 independent experiments.

To evaluate the in vivo side effects of GalNAc-siRNAs, off-target effects, immunogenicity reactions, and toxicity were tested. Firstly, six genes that had similarity greater than 95% with siX61 including *Sulf2*, *Hectd2*, *Irs2*, *Smg1*, *Gng12*, and *Mllt10* were detected by qPCR. The results showed that there was no significant inhibition in relative mRNA expression of these genes by GalNAc-siX61 (Figure 10A), indicating that GalNAc-siRNA had no off-target effects in vivo. Secondly, the serum levels of pro-inflammatory cytokines including TNFα, IL6, and IFNα were determined by ELISA. The results indicated that the levels of pro-inflammatory in the GalNAc-siX61 group mice did not change compared with the saline group mice (Figure 10B).

**Figure 11:**
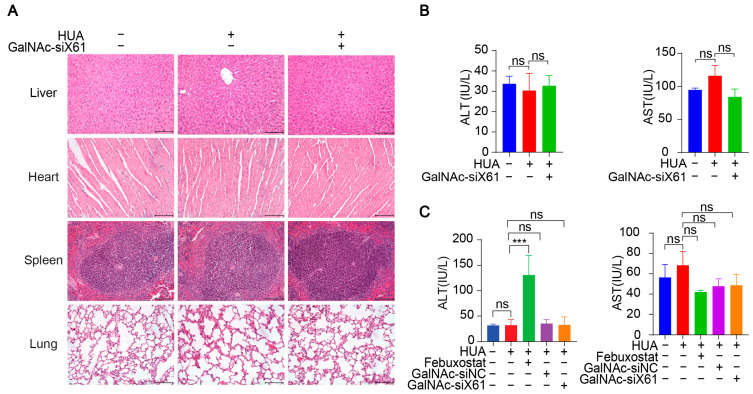
GalNAc-siRNAs showed no notable toxicity in vivo. (**A**) Representative images of HE staining of livers, hearts, spleens, and lungs from *hXDH* mice in the NC, hyperuricemia, and GalNAc-siX61 groups. Scale bar = 100 μm. (**B**) No obvious changes were found in aspartate aminotransferase (AST) and alanine aminotransferase (ALT) levels after administration of GalNAc-siX61 in *hXDH* mice. (**C**) The concentrations of ALT and AST in plasma from *WT* mice treated with febuxostat and GalNAc-siX61 were tested. ns, *p* > 0.05; *** *p* < 0.001. *n* = 3 independent experiments.

In addition, various organs were harvested from *hXDH* mice on the 21st day after multiple doses of GalNAc-siX61 and were stained with hematoxylin and eosin (HE). No notable histological differences were found in the tissues from the liver, heart, spleen, or lung between the NC-, HUA-, and GalNAc-siRNAs-treated groups, suggesting no notable toxicity (Figure 11A). The concentrations of AST and ALT in plasma were tested to assess liver function, showing no hepatotoxicity of GalNAc-siRNAs administration (Figure 11B). What is more, the ALT level of the febuxostat group increased markedly in *WT* mice (Figure 11C), indicating the liver damage was induced by febuxostat, which was consistent with previous reports [38]. The data indicated that targeting liver *Xor* with GalNAc-siRNAs was a promising strategy for hyperuricemia therapy with high effectiveness and safety. 

## 4. Discussion

XOR is a key, rate-limiting enzyme in uric acid synthesis. Drugs targeting XOR, such as febuxostat and allopurinol, have been widely used in clinical treatment of hyperuricemia. Therefore, XOR has been an effective target for hyperuricemia therapy and is of great interest. It was previously thought that the liver is the main site of uric acid synthesis, and the serum uric acid level was greatly reduced in liver-specific *Xdh* knockout mice [14,39]. Only reducing hepatic XOR expression prevents severe renal failure accompanied by accumulation of crystals and triglycerides in renal tubules and interstitial fibrosis caused by whole-body knockout of the *Xdh* gene [40]. However, clinical trials of Alnylam’s GalNAc-siRNA, which specifically targets liver *XOR*, have been stopped for unknown reasons. Therefore, whether liver XOR is the main synthetase of uric acid in the body and whether targeting only liver *XOR* is sufficient for the treatment of hyperuricemia remain controversial.

In addition to the liver, XOR is expressed in many tissues, including the muscle, kidney, intestine, WAT, BeAT, and BAT. However, GalNAc-siX61, which specifically targets liver XOR, significantly reduced plasma uric acid levels and alleviated kidney damage caused by high uric acid levels in various hyperuricemia mouse models. These results suggested that the liver is the main site of uric acid formation and that targeting liver XOR can effectively treat hyperuricemia.

After *Uox* knockout, only uric acid levels in the liver, brown adipose tissue, and kidney increased, which indicated that these tissues were the primary sites where uric acid is metabolized. We found that BAT is also involved in uric acid synthesis and metabolism in the body. It has been reported that high levels of uric acid inhibit BAT thermogenic capacity through the regulation of AMPK [41], but the physiological and pathological significance of the uric acid synthesis pathway in BAT needs to be further studied. In fact, XOR is widely distributed and plays different roles in various tissues [42]. XOR contributes to apocrine lipid secretion in the breast [43], counteracting the growth of pathogens [44] in milk and supporting blood pressure in the kidney [42]. Specifically, targeting liver XOR may prevent adverse effects caused by interfering with XOR functions in other tissues.

Hyperuricemia is often associated with kidney injury [45], but the causal relationship is unclear. We found that in *Uox* knockout mice, the levels of uric acid in the liver and brown fat increased by less than threefold, but the levels of uric acid in the kidneys increased dramatically. This indicated that uric acid was quickly transferred to the kidney for excretion after synthesis. When the increase in uric acid level exceeds the excretion capacity of the kidney, a large amount of uric acid accumulates in the kidney. This may also explain why the kidney is more susceptible to hyperuricemia than other tissues and organs.

In addition, urate crystals were not observed in kidneys of hyperuricemia mice, and we found that the level of soluble uric acid was associated with renal damage in vivo. These data suggested that uric acid may also lead to renal damage in a urate stone-independent manner. Several studies have reported that uric acid induced renal tubule epithelial cells and endothelial dysfunction and interstitial fibrosis [46], and activated inflammation in the kidney through oxidative stress, epithelial-to-mesenchymal transition, regulating NLRP3 inflammasome-mediated IL-1β secretion [47], reducing levels of SUMO-PPARγ, etc. [48]. XOR catalyzes the oxidative hydroxylation of hypoxanthine to xanthine to uric acid, with the accompanying production of two reactive oxygen species (ROS), superoxide anion (O_2_^−^) and hydrogen peroxide (H_2_O_2_). O_2_^−^ can react with NO at a relatively fast speed, reducing NO bioavailability, thus inducing endothelial dysfunction. O_2_^−^ and H_2_O_2_ can be converted to peroxynitrate (ONOO^−^), hydroxyl anion (OH^−^), and hypochorous acid (HOCl), which damage proteins, carbohydrates, nucleic acids, and other cell components, bringing toxicity to cells [38,49]. Therefore, the liver XOR inhibition may alleviate hyperuricemia through the reduction in both uric acid and ROS.

While siRNA demonstrates high efficacy in suppressing target gene expression post-transcriptionally, its in vivo delivery encounters substantial challenges. These include difficulty in escaping endosomes to reach the cytosol, susceptibility to renal clearance, and so on [50]. In order to enhance the delivery efficiency of siRNA, several delivery systems have been developed. GalNAc was a novel, effective, and specific delivery systems to the liver. There have been several successful examples of the medicine in clinical use [51]. Givosiran, the second siRNA drug approved by the FDA in October 2019, was followed swiftly by three additional medicines: lumasiran (approved in 2020), inclisiran (approved in 2021), and vutrisiran (approved in 2022). All of these siRNA drugs are GalNAc–siRNA conjugates [52,53,54,55]. For example, inclisiran can inhibit the synthesis of protein convertase subtilisin-kexin type 9 (PCSK9), thus lowering the level of low-density lipoprotein (LDL) and helping people with atherosclerotic cardiovascular disease (ASCVD), ASCVD risk-equivalents, or heterozygous familial hypercholesterolemia (HeFH). Therefore, the GalNAc-siRNA platforms will become more and more popular for liver-related disease therapy in the future.

As a method of administration, the s.c. route has been commonly employed for administering vaccines, insulin, heparin, biological agents, and medications with high molecular weights [51,56]. Subcutaneous administration offers advantages, combining ease of use, minimal effects on patient mobility, and reduced risks of thrombosis and infections compared to other routes [57]. However, subcutaneous drug delivery also has challenges. Because the injection volume is small, the dose should not be too large. Therefore, the drug needs to be effective at lower doses or the drug can be made into a high concentration formula and is stable at high concentrations. For GalNAc-siRNA, it is highly efficient and stable, so it can meet the requirements of subcutaneous administration. In fact, GalNAc-siRNA is mainly administered by subcutaneous injection in clinic.

## 5. Conclusions

To summarize, this study suggested a new method in the treatment of hyperuricemia with little side effect. The pivotal role of hepatic xanthine oxidoreductase (XOR) in uric acid synthesis was identified, and the specific targeting of XOR within the liver demonstrated a mitigating effect on renal damage induced by elevated uric acid levels in hyperuricemia mice. This study provided a novel strategy and gave inspiration to develop new drugs targeting the liver for treating hyperuricemia.

## Data Availability

All data will be made available by the corresponding author upon reasonable request.
